# Acceptance and Commitment Therapy in Daily Life Training: A Feasibility Study of an mHealth Intervention

**DOI:** 10.2196/mhealth.5437

**Published:** 2016-09-15

**Authors:** Tim Batink, Jindra Bakker, Thomas Vaessen, Zuzana Kasanova, Dina Collip, Jim van Os, Marieke Wichers, Inez Germeys, Frenk Peeters

**Affiliations:** ^1^ Department of Psychiatry and Psychology Maastricht University Medical Centre Maastricht University Maastricht Netherlands; ^2^ U-Center Epen Netherlands; ^3^ Center for Contextual Psychiatry Department of Neuroscience KU Leuven Leuven Belgium; ^4^ Institute of Psychiatry Department of Psychosis Studies King’s College London London United Kingdom; ^5^ University Medical Center Groningen, Interdisciplinary Center Psychopathology and Emotion Regulation Department of Psychiatry University of Groningen Groningen Netherlands; ^6^ Virenze RIAGG Maastricht Netherlands

**Keywords:** mHealth, behavior change, daily life intervention, acceptance and commitment therapy, experience sampling

## Abstract

**Background:**

With the development of mHealth, it is possible to treat patients in their natural environment. Mobile technology helps to bridge the gap between the therapist’s office and the “real world.” The ACT in Daily Life training (ACT-DL) was designed as an add-on intervention to help patients practice with acceptance and commitment therapy in their daily lives. The ACT-DL consists of two main components: daily monitoring using experience sampling and ACT training in daily life.

**Objectives:**

To assess the acceptability and feasibility of the ACT-DL in a general outpatient population. A secondary objective was to conduct a preliminary examination of the effectiveness of the ACT-DL.

**Methods:**

An observational comparative study was conducted. The experimental group consisted of 49 patients who volunteered for ACT-DL, and the control group consisted of 112 patients who did not volunteer. As part of an inpatient treatment program, both groups received a 6-week ACT training. Participants went home to continue their treatment on an outpatient basis, during which time the experimental group received the 4-week add-on ACT-DL. Acceptability and feasibility of the ACT-DL was assessed weekly by telephone survey. Effectiveness of the ACT-DL was evaluated with several self-report questionnaires ( Flexibility Index Test (FIT-60): psychological flexibility, Brief Symptom Inventory: symptoms, Utrechtse Coping List: coping, and Quality of life visual analog scale (QoL-VAS): quality of life).

**Results:**

More than three-quarters of the participants (76%) completed the full 4-week training. User evaluations showed that ACT-DL stimulated the use of ACT in daily life: participants practiced over an hour a week (mean 78.8 minutes, standard deviation 54.4), doing 10.4 exercises (standard deviation 6.0) on average. Both ACT exercises and metaphors were experienced as useful components of the training (rated 5 out of 7). Repeated measures ANCOVA did not show significant effects of the ACT-DL on psychological flexibility (*P*=.88), symptoms (*P*=.39), avoidant coping (*P*=.28), or quality of life (*P*=.15).

**Conclusions:**

This is the first study that uses experience sampling to foster awareness in daily life in combination with acceptance and commitment therapy to foster skill building. Adherence to the ACT-DL was high for an intensive mHealth intervention. ACT-DL appears to be an acceptable and feasible mHealth intervention, suitable for a broad range of mental health problems. However, short-term effectiveness could not be demonstrated. Additional clinical trials are needed to examine both short-term and long-term effects.

## Introduction

### Background

Over the last decade, the field of psychological treatment has seen the emergence of third generation behavioral therapies [1]. These third wave therapies focus on how to relate in a more workable way to difficult thoughts and feelings rather than trying to change them. One of these therapies is acceptance and commitment therapy (ACT). Instead of focusing on the content, the person focuses on the function of difficult experiences and the context in which they occur [2]. Here, we examine a novel strategy designed to implement ACT interventions in the daily lives of patients, thereby enhancing the usefulness and effect of the treatment.

### Acceptance and Commitment Therapy

**Figure 1:**
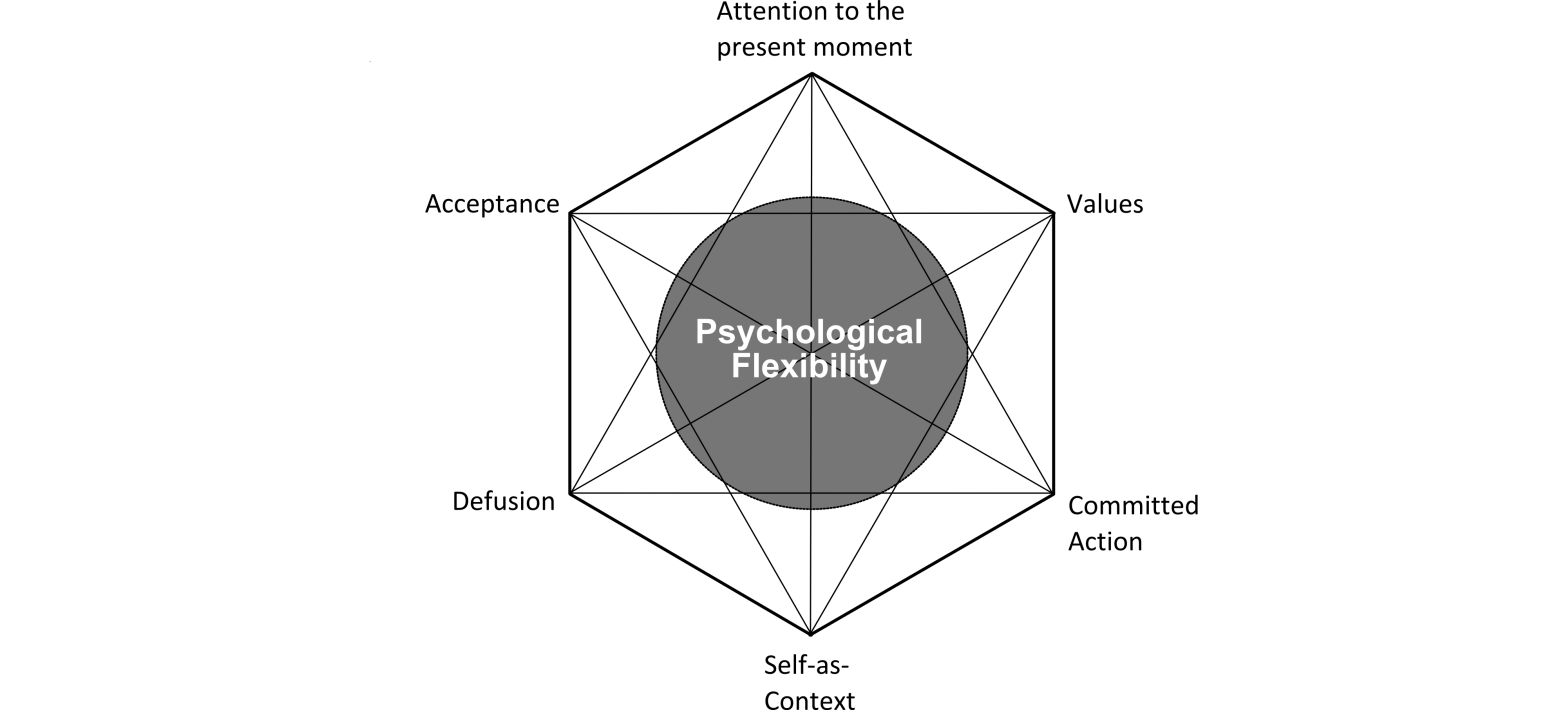
The ACT hexaflex with the 6 core processes.

ACT is a form of psychotherapy positioned within the third wave behavioral therapies. ACT does not primarily focus on symptom reduction but teaches patients to deal with their challenging experiences in such a way that they can behave according to their values [2]. The ACT model consists of six core processes that are closely interlinked (Figure 1). *Acceptance* teaches patients to pay attention to unpleasant feelings instead of trying to get rid of them. *Defusion* helps patients to recognize thoughts for what they are: cognitions they can distance themselves from rather than truths they have to react on. The *self as context* helps patients realize they are more than their thoughts, emotions, and self-image; there is also an observer who is having these experiences. Patients also learn to focus their *attention to the present moment*—to be aware of their internal and external environment. *Values* helps patients refocus on the things that really matter to them, and *committed action* encourages them to start investing in their personal values again. Together, these core processes form psychological flexibility, the ability to deal with challenging experiences in a flexible way while continuing to act based on one’s values [3,4]. Since ACT is an experiential form of therapy, many exercises are used (learning by experiencing). The exercises also support skill-building by patients. Metaphors are also frequently applied in this form of therapy to validate the patient’s experience, create awareness of the situation, and introduce new approaches to handle the situation [5].

The American Psychological Association has included ACT in its register of research-supported psychological treatments for depression, mixed anxiety, obsessive-compulsive disorder, psychoses, and chronic pain. A recent meta-analysis from A-Tjak and colleagues [6] showed that ACT is an effective intervention for treating depression, anxiety disorders, addiction, and somatic health problems. The evidence thus suggests transdiagnostic effectiveness of ACT.

Although it is most valuable to acquire ACT skills during therapy sessions, it is equally important to get ACT out of the therapist office and into the daily life of the patient. This helps the patient to generalize ACT skills to different situations and different challenges, changing behavioral patterns at home. Put shortly: learning to apply ACT in their daily lives.

### Experience Sampling Method

The experience sampling method (ESM) is an ambulatory assessment method that consists of multiple assessments per day, at random times, asking patients to complete a brief questionnaire about experiences, environments, and activities in-the-moment (ecological validity) [7]. By measuring in daily life, ESM takes into account the importance of the context in which experiences and behavior occur and also provides information regarding this context [8].

From an ACT perspective, ESM not only represents a suitable ecologically valid assessment method but also facilitates the ACT processes *attention to the present moment* and *self as context*, which together represent the core pillar “being aware.” Completing multiple assessments at random moments throughout the day promotes awareness of both internal and external experiences, as well as insight into the relationship between experiences and context [9].

### mHealth

Mobile Health (mHealth) is a relatively recent technological development and can be defined as the use of mobile devices such as personal digital assistants (PDAs), mobile phones, and tablets to promote health [10,11]. In February 2016, 72% of adults in the United States and 60% to 71% of adults in Western Europe owned a mobile phone [12]. Mobile devices can assess and intervene at any time or place in the personal context of the user (online and offline). Therefore, they are an ideal medium for personal ecological assessment and intervention. ESM has already successfully found its way in mHealth [13-18], showing its potential in the treatment of depression [19] and anxiety [20]. Similarly, the first mHealth ACT interventions are showing promising results for chronic medical conditions [21], stress [22,23], and smoking cessation [24].

### Objectives

The ACT in Daily Life training (ACT-DL) focuses on daily life monitoring and ACT training in context, combining ACT techniques, ESM, and mobile technology (see Multimedia Appendix 1 for example). The goal of the intervention is to stimulate patients to practice ACT skills in their daily life after completing a regular ACT training. The primary objective was to assess the ACT-DL with respect to feasibility and acceptability in a heterogeneous population of patients with a mental health disorder. The secondary objective was to conduct a preliminary examination of the effectiveness of ACT-DL.

## Methods

### Participants

Participants were recruited in an inpatient mental health care facility in Epen, the Netherlands, between June and November 2013. The center provides treatment for common mental problems like major depression, anxiety, and substance use disorder. All patients received a flyer about the study with information about the aim and set-up of ACT-DL, duration of the training, and estimated time investment. Inclusion criteria were intentionally kept broad; all consecutive referrals in the study period could participate in the study. There were two exclusion criteria: deviation from the length of inpatient stay (standard 7-week program) and participation in another study within the treatment facility. The control group consisted of all other patients who participated in the full treatment program (7-week inpatient and 7-week outpatient treatment) between 2013 and 2014 and completed all the required measures. Due to the clinical setting, it was impossible to randomize participants into two conditions and therefore an observational design was chosen.

The initial aim for the feasibility study was to include a minimum of 20 participants (suggested by sample size calculation, Cohen *d*=1.8, alpha error probability = .05, power = .95, allocation ratio N2/N1=1). Given the enthusiasm for participation, 49 participants were included in the experimental intervention (mean age 45.7 years, standard deviation [SD] 10.0 years) and 112 participants composed the control group (mean age 47.5 [SD 12.4] years).

### Sample Characteristics

Both the ACT-DL and the control group were heterogeneous with regard to age, level of education, and diagnosis on *Diagnostic and Statistical Manual of Mental Disorders* (Fourth Edition) (DSM-IV-TR), Axis I and Axis 2 (Table 1). There were no significant differences in demographics between the ACT-DL and the control group with the exception of gender (*P*=.02). A total of 8 participants dropped out after the first week, 3 after the second week, and 1 after the third week. Of the 49 participants, 37 (76%) completed the full 4-week ACT-DL. The completers did not deviate significantly on demographics or baseline measures from the noncompleters. Additionally, because of the qualitative nature of the evaluation by telephone (participants did not always give a clear answer on every question), missing data points were present in the dataset (20%) and assumed to be missing at random. An intention-to-treat analysis was used.

### Procedures

The Medical Ethics Committee of Maastricht University Medical Centre approved the study procedures, and all participants provided written informed consent. All participants underwent a 7-week inpatient treatment program that started with a 1-week diagnostic phase including structured interviews in which DSM-IV-TR axis I (Mini-International Neuropsychiatric Interview, or MINI [25]) and axis II disorders (Structured Interview for DSM-IV-TR Personality Disorders [26]) were assessed. Participants also completed a range of pretreatment questionnaires (T1). After the diagnostic week, participants followed an intensive 6-week treatment program (psychoeduction, cognitive-behavior therapy, ACT, mindfulness, group psychotherapy, creative therapy, relapse prevention, and vitality management). One of the main components of the program was an ACT group. This was a weekly, 90-minute group therapy session (10-12 members) guided by a structured treatment manual, during which one of the 6 ACT core components was targeted each week (handouts were provided). Throughout the 6 sessions, the metaphors and exercises that were going be used in the ACT-DL during the outpatient phase were discussed. An experienced ACT therapist conducted the weekly sessions.

After the 6-week inpatient program, participants completed the posttreatment questionnaire battery (T2). During the last week of inpatient treatment, participants who volunteered for the ACT-DL received a 60-minute briefing on how the training would be implemented in their daily lives. At this point, the participants of the ACT-DL training received the PsyMate digital devices with the ACT-DL program. Participants were asked to keep the PsyMate with them at all times during the training days but not let this interfere with their daily lives.

**Figure 2:**
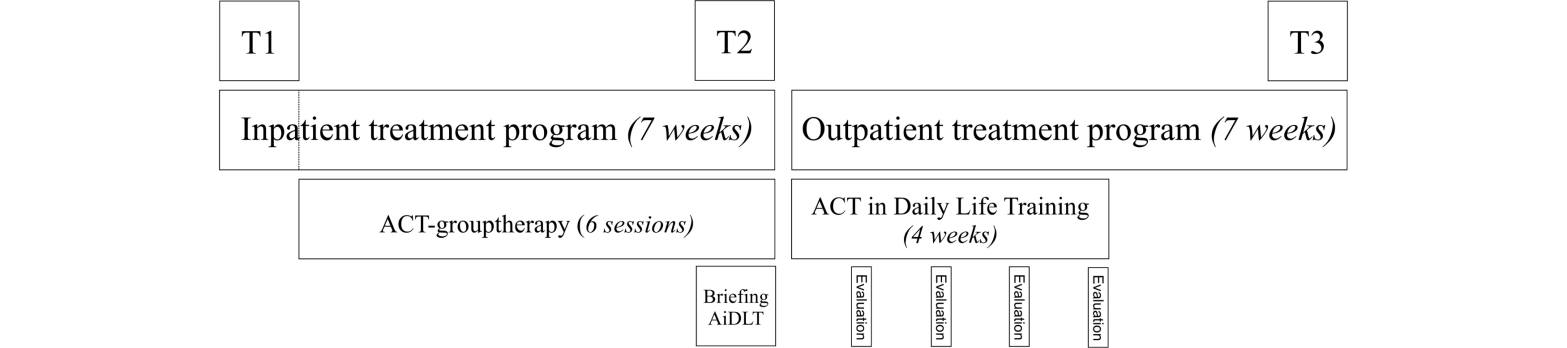
Timeline of the procedure for the experimental group. ACT: acceptance and commitment therapy.

Treatment was then continued for a 7-week outpatient phase to which a 4-week ACT-DL was added in the experimental group. ACT-DL participants started with the 4-week training as soon as they arrived home. At the end of each week of training, participants were contacted by a member of the research staff for a semistructured interview (approximately 15 minutes) on their experiences of that week. The evaluation of the last training week was more elaborate and was also used for debriefing (approximately 30 minutes). The answers to the Likert-scale questions were noted, and the answers to the open-ended questions were transcribed. At the end of the outpatient treatment phase, participants completed the postoutpatient questionnaire battery (T3, Figure 2) and returned the PsyMates with the ACT-DL program.

**Table 1:** Demographic characteristics of participants in the intervention (n=49) and control (n=112) groups.

		ACT-DL^a^	Control	*P* value
Age, years, mean (SD)		45.7 (10.0) min 23-max 65	47.5 (12.4) min 22-max 68	.38
Gender, n (%)				.02
	Male	17 (35)	62 (55)	
	Female	32 (65)	50 (45)	
Education level, n (%)				.15
	Primary	—	1 (1)	
	Secondary	21 (43)	51 (46)	
	Undergraduate	17 (35)	21 (19)	
	Graduate	11 (22)	35 (31)	
	Other	—	4 (4)	
Main diagnosis on Axis I (DSM-IV-TR-TR^b^), n (%)				.63
	Anxiety	6 (12)	20 (18)	
	Mood	26 (53)	44 (39)	
	Somatoform	4 (8)	9 (8)	
	Substance	11 (22)	31 (28)	
	Other	2 (4)	8 (7)	
Additional diagnosis on Axis II (DSM-IV-TR-TR) , n (%)				.10
	None	36 (74)	90 (80)	
	Cluster A	—	—	
	Cluster B	6 (12)	4 (4)	
	Cluster C	5 (10)	8 (7)	
	Other	2 (4)	10 (9)	

^a^ACT-DL: ACT in Daily Life.

^b^DSM-IV-TR-TR: *Diagnostic and Statistical Manual of Mental Disorders*(Fourth Edition, Text Revision)

### Intervention: ACT in Daily Life Training

#### Design of ACT in Daily Life

The ACT-DL is a fully automated mobile ACT intervention delivered by a PDA-like device, the PsyMate (see Myin-Germeys et al [27] for a global description). This mHealth intervention assists participants to practice with ACT skills in their daily lives. The goal of the training is to help participants integrate ACT skills in their daily life, thus improving psychological flexibility and, ultimately, quality of life. The ACT-DL has a duration of 4 weeks during which participants receive training for 3 consecutive days each week—Thursday, Friday, and Saturday—to get a cross-section of the week (approximately 1 hour per day). The ACT-DL is not a stand-alone intervention. Participants need to be familiar with ACT (prior therapy/training) to be able to benefit from this add-on intervention. The ACT-DL consists of two main components: daily monitoring via experience sampling and ACT training in the personal environment. The ACT-DL uses persuasive techniques such as tunnelling (leading users through a predetermined sequence of actions), self-monitoring, and reminders.

#### PsyMate

**Figure 3:**
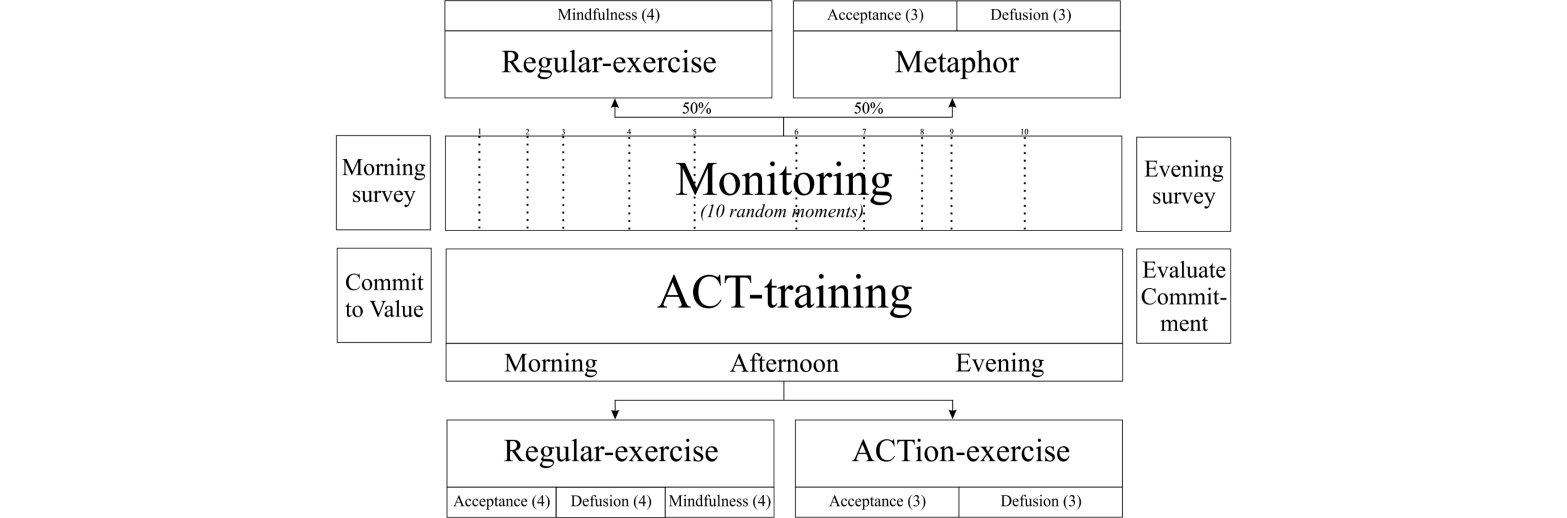
Schematic overview of the ACT in Daily Life Training (ACT-DL). The ACT-DL consists of two main components: monitoring and ACT training.

The PsyMate is a PDA-like device that provides the ACT-DL. It facilitates both the monitoring in daily life as a pocket diary and the ACT training by providing 18 different ACT exercises and 6 ACT metaphors as described above. The mode of delivery of the exercises is text-based; metaphors are offered as an illustration. Since it is a mobile device, it allows participants to practice with ACT in their daily lives. Figure 3 provides a schematic overview of the ACT-DL.

#### Monitoring

The ACT-DL uses daily life monitoring to foster awareness of one’s mental state, as well as the impact of the context on that state. Between 7:30 AM and 10:30 PM, the PsyMate beeps randomly 10 times, prompting the participant to complete a brief self-report questionnaire about current symptoms (affect and cognition), activity, company, and whereabouts. Furthermore, participants are asked to complete a brief morning questionnaire and evening questionnaire, appraising their day and quality of sleep. Monitoring was also restricted to the three ACT-DL training days.

#### ACT Training

The ACT-DL focuses on four core components of ACT: acceptance, defusion, mindfulness, and committed action. Two types of ACT exercises are available via the PsyMate during the ACT-DL: regular exercises and ACTion exercises.

The regular exercises are always applicable because they focus on general ACT skill training independent of current thought, feelings, or situations. The exercises on acceptance, defusion, and mindfulness are made available on demand (4 exercises per component). Participants are required to carry out at least 3 exercises of their choice per training day (morning, afternoon, evening). Also, mindfulness-related exercises are offered after 5 out of 10 self-assessments (signal-contingent).

The ACTion exercises are specifically designed to be applicable in distressing situations. Whenever a participant has an unpleasant thought or feeling, they can activate one of the ACTion exercises (event-contingent) to deal with those distressing experiences in an ACT-consistent manner. Both acceptance and defusion ACTion exercises are available (3 exercises per component, see Textbox 1).

**Example of an ACTion exercise.**

Acceptance exercise: Opening up.

- Unpleasant feelings are showing up for you right now.
- See if you can open up to them, allowing these feelings to be there.
- Explore what there is to experience—what goes through your mind?
- Can you stay present with these difficult feelings and keep in touch with them?
- Do these feelings remain the same, or do they change?
- Are they getting heavier, lighter, do they remain the same, or do they fluctuate?
- See if you can continue giving some space to these unpleasant feelings for a while instead of trying to control them or trying to get rid of them.

In order to integrate committed action in the ACT-DL, at the end of each morning questionnaire, participants have to choose a personal value they want to invest in that day. Following the evening questionnaire, participants have to evaluate whether they had invested in their chosen value of the day (interval-contingent).

**Figure 4:**
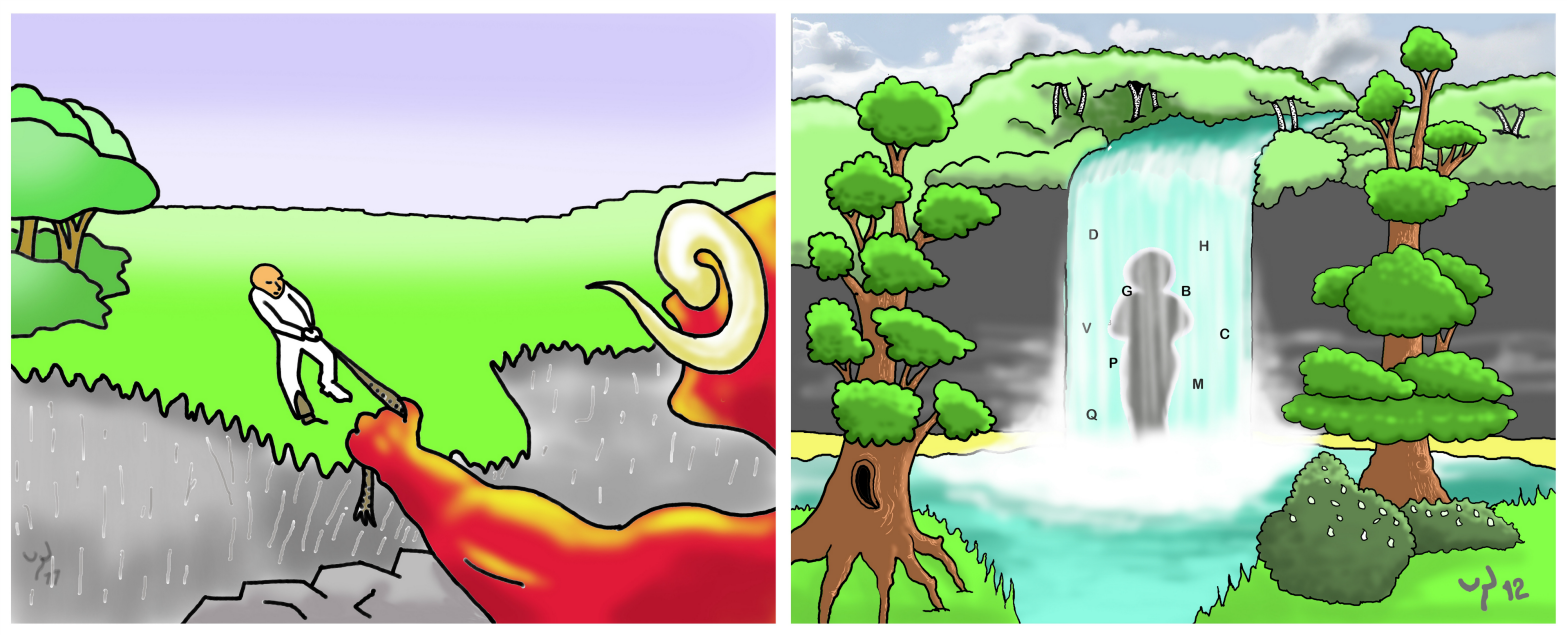
Examples of illustrated ACT metaphors. (Left, acceptance metaphor: tug of war. Try to stop your attempts of solving your pain by winning the war with it; try to let go of the rope. Right, defusion metaphor: waterfall. Instead of getting carried away by your stream of thoughts, take a step back and observe them.).

In addition to the ACT exercises, illustrated ACT metaphors are used in the training (Figure 4). These illustrated metaphors serve as a reminder/cue to reactivate important ACT concepts learned during previous ACT training sessions (without needing to explain the concept in detail again). Both metaphors for acceptance and defusion can be accessed on demand (3 metaphors per component). The ACT metaphors are also offered after 50% of the self-assessment moments during the day, alternating with the mindfulness exercises (after 5 out of 10 self-assessments [signal-contingent]).

### Measures

#### Usage and User Experiences

During the 4-week intervention period of the ACT-DL training, participants were contacted once a week by phone by a member of the research team to evaluate the previous week’s training. This evaluation was conducted via a semistructured interview during which participants were asked to evaluate the usability of the PsyMate, the ACT metaphors, and the ACT exercises of that week. Open-ended questions (eg, “How did your practice go last week?”) and questions using a 7-point Likert scale (eg, “The ACT exercises were useful; 1 = not at all, 7 = very much") were used. Also usage of the exercises and metaphors was evaluated (eg, “How many ACT exercises did you on average perform per day?”). After the last week, a more elaborate evaluation was conducted.

#### Psychological Flexibility

The Flexibility Index Test (FIT-60) is a self-report questionnaire that measures psychological flexibility and its six underlying ACT components: acceptance, defusion, self as context, present moment, values, and committed action [26]. The FIT-60 consists of 60 items that are scored on a 7-point Likert Scale. The FIT-60 has good psychometric properties [28,29].

#### Coping Skills

The Utrechtse Coping List (UCL) is a 47-item self-report questionnaire that measures seven coping styles: actively addressing, palliative reacting, avoiding, seeking social support, passive reacting, expression of emotions, and reassuring thoughts [30]. Only the subscale avoiding was used for this study. The internal consistency of the UCL is moderate to good. Construct validity and predictive validity are sufficient [31].

#### Psychological Symptoms

The Brief Symptom Inventory (BSI) provides an overview of symptoms and their intensity at a specific point in time [32]. The BSI consists of 53 items which are scored on a 5-point Likert scale. Besides a total symptom rating, the BSI indexes symptoms on 9 specific dimensions: somatization, obsessive-compulsive, interpersonal sensitivity, depression, anxiety, hostility, phobic anxiety, paranoid ideation, and psychoticism. The internal reliability and test-retest are sufficient. Furthermore, the inventory is sensitive to treatment effects [33,34].

#### Quality of Life

Quality of life was assessed with the question “How happy do you feel at this moment?” and scored on a 10-point visual analog scale (QoL-VAS). This question is used as a proxy for living a valued, committed life. The Dutch QoL-VAS has good construct validity [35].

Participants completed the self-report questionnaires before starting the inpatient treatment (T1: preinpatient), after the inpatient treatment (T2: postinpatient), and after the outpatient treatment, during which the experimental group also received the ACT-DL (T3: postoutpatient).

### Statistical Analyses

Feasibility and acceptability of the ACT-DL were assessed with descriptive statistics. The open-ended answers of the user evaluation were analyzed via a conventional qualitative content analysis method (codes were derived from the verbatim transcripts and merged into a coding scheme). The effectiveness of the ACT-DL on ACT skills, coping, quality of life, and clinical symptoms were analyzed using SPSS version 22.0 statistical software (IBM Corp) with several analyses of covariance (ANCOVAs, level of significance was adjusted for the number of hypotheses with a Bonferroni correction; *P*=.05/4: *P=*.01). Interaction effects were tested with a repeated measures ANCOVA.

## Results

### Acceptability and Feasibility of ACT in Daily Life

**Figure 5:**
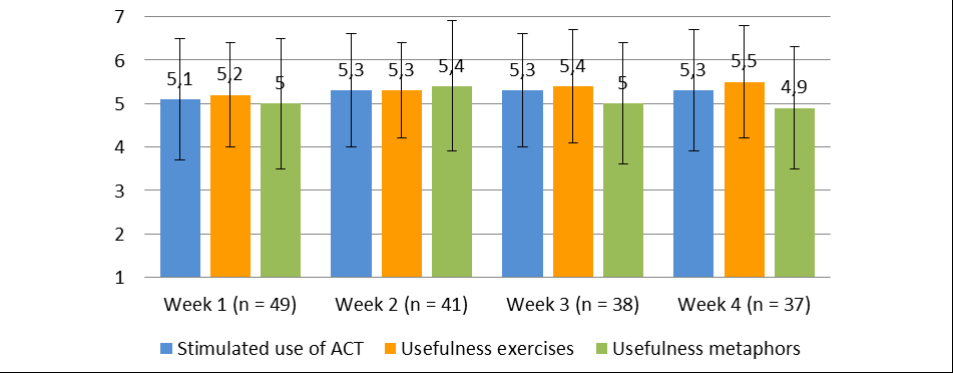
User experience of the ACT in Daily Life Training (ACT-DL).

The semistructured evaluation via telephone of the ACT-DL focused on user experience. These evaluations revealed that the training was rated positively, it stimulated the use of ACT, and the ACT exercises and metaphors were rated as useful (Figure 5). The ratings did not significantly change over the four assessment periods, assessed with a repeated measures analysis of variance regarding use of ACT (*F*_3,42_=0.063, *P*=.98), usefulness of the exercises (*F*_3,39_=0.856, *P*=.47), and usefulness of the metaphors (*F*_3,42_=0.788, *P*=.51). The ACT exercises and ACT metaphors were rated equally useful (*t*_129_=−1.453, *P*=.15). Participants were asked to keep track of the number of exercises they activated and how much time they spent on the training. Participants activated on average 8 to 13 exercises per week (mean 10.4, SD 6.0) in addition to the 15 exercises that were prompted that week and spent between 69 and 85 minutes (mean 78.8, SD 54.4) on the ACT training each week. The use of the ACT-DL was consistent over time, with a minor decline in use of exercises during week 3 (mean −2.5).

Participants responded with an average of 5.8 (SD 1.2) out of 7 on the question whether they would recommend the ACT-DL to others. Remarks from the participants during the debriefing about the training were, for example: “It helped me practice more with ACT than I would normally do,” “It kept me aware of myself,” and “I want to continue with this training.”

When asked for suggestions for improvement of the ACT-DL, the following three considerations were proposed most commonly by the participants. The first one was the desire for more ACT exercises and more variations within these exercises (more topics, 7x). In addition, it was suggested to reduce the number of ACT metaphors that were offered each day (7x). A final suggestion was the preference for having auditory rather than visual awareness exercises (4x).

The same question was also presented to the two research assistants who conducted the close to 200 semistructured interviews by phone. An important observation both interviewers made was that some participants seemed to have had difficulty discerning between the regular ACT exercises and the ACTion exercises (which were specifically designed to help participants deal with momentary negative thoughts and feelings).

### Effectiveness of ACT in Daily Life

The effectiveness of the ACT-DL was assessed with self-report questionnaires. Three assessment moments were used (Table 2): preinpatient (T1), postinpatient (T2), and postoutpatient (T3). The effectiveness of the ACT-DL was assessed by comparing results between T2 and T3. Gender was taken into account as a possible confounder. As can been seen in Table 2, generally, the direction of the effects between T1 and T2 shows improvement (decline of symptoms, increase in skills), whereas the direction of the effects between T2 and T3 shows a slight deterioration (rise of symptoms, decline in skills).

**Table 2:** Results of self-report questionnaires at different assessment time points for participants in the intervention (n=49) and control (n=112) groups in ACT in Daily Life Training (ACT-DL).

	ACT-DL Mean (SD)			Control Mean (SD)		
	T1^a^	T2^b^	T3^c^	T1^a^	T2^b^	T3^c^
FIT-60^d^	177.4 (33.8)	206.2 (50.3)	197.7 (53.0)	181.0 (39.4)	215.4 (52.6)	208.8 (58.0)
BSI^e^	1.40 (.65)	.70 (.52)	1.00 (.72)	1.31 (.62)	.67 (.57)	.85 (.72)
UCL (avoiding)^f^	18.2 (3.5)	17.7 (3.5)	17.8 (3.3)	17.8 (3.5)	17.0 (3.0)	17.3 (3.4)
QoL-VAS^g^	2.3 (2.3)	5.8 (2.5)	4.4 (3.0)	2.8 (2.2)	5.8 (2.4)	5.3 (2.9)

^a^T1: Preinpatient.

^b^T2: Postinpatient.

^c^T3: Postoutpatient.

^d^FIT-60: Flexibility Index Test.

^e^BSI: Brief Symptom Inventory.

^f^UCL: Utrechtse Coping List.

^g^QoL: Quality of Life—Visual Analog Scale.

Results show that the ACT-DL group and the control group did not differ significantly from each other at the preinpatient (T1) and the postinpatient assessment (T2). Also at the post outpatient assessment (T3), the groups did not differ significantly on psychological flexibility (*F*_1,152_=1.346, *P*=.25), symptoms (*F*_1,153_=1.094, *P*=.30), avoidant coping (*F*_1,148_=.153, *P*=.70) and quality of life (*F*_1,147_=0.569, *P*=.11). Furthermore, time (T2-T3) did not have a significant effect on these four outcomes for the ACT-DL-group (*P*=.03 to *P*=1.00). The repeated measures ANCOVA also showed no significant interactions between treatment group and time (T2-T3) on psychological flexibility (*F*_1,152_=0.023, *P*=.88), symptoms (*F*_1,153_=0.748, *P*=.39), avoidant coping (*F*_1,147_=1.184, *P*=.28), or quality of life (*F*_1,146_=2.053, *P*=.15). Treatment dose effect of the ACT-DL has been examined with Pearson correlations, showing a weak (*r*=.040 to .210) and nonsignificant (*P*=.26 to .86) association between dosage, defined as self-reported information on number of executed exercises during the ACT-DL and minutes of time spent on those exercises, and treatment effect. Finally there were no significant differences between diagnoses (determined with the MINI) on treatment effect regarding psychological flexibility of the ACT-DL (*F*_1,44_=0.005, *P*=.95).

## Discussion

### Principal Findings

The primary objective of this study was to assess the feasibility and acceptability of the ACT-DL in a heterogeneous mental health population. Due to enthusiasm for participation, the recruitment aim was more than doubled (20 to 49). Adherence to treatment was adequate given the intensity of the mHealth intervention with 76% of participants completing the full 4-week training. Other mHealth studies reported dropout rates as high as 50% [36], and even higher attrition rates were reported with Web-based interventions [37]. A systematic review from Kelders et al [38] has identified important variables that could explain why the adherence to the ACT-DL was relatively high: the intervention used persuasive techniques (tunnelling, self-monitoring, and reminders); participants had frequent contact with the researchers; and the duration of the training was relatively short (4 weeks), which has been shown to produce higher adherence than interventions with a longer duration [39]. Taken together, the ACT-DL seems to be a feasible mHealth treatment. User evaluations showed that the program stimulated the use of ACT in daily life; participants practiced over an hour a week, doing 10 exercises on average. Both ACT exercises and metaphors were experienced as useful components of the training. Also participants would highly recommend the training to others. These results also suggest high acceptability of the ACT-DL. These findings are in line with previous studies [21-24], showing that it is feasible to deliver ACT via mHealth.

User evaluations showed that the acceptability of ACT-DL could be enhanced by extending the number of available ACT exercises. Auditory awareness exercises could also increase treatment adherence. Although ACT metaphors were generally appreciated, the frequency should be limited (once or twice a day). A further recommendation was that the tool should be simple in its design and functions (distinguishing between regular ACT exercises and ACTion exercises seemed to be unclear for some participants). These results correspond with and add to recommendations of a similar nature by Ahtinen and colleagues [40], who stressed easy-to-do daily life exercises and guiding participants gently through but not restricting choice in exercises.

A revised version of the ACT-DL, based on lessons learned from this study, will be used in future research (H, Steinhart, MSc, unpublished data, 25-2-2016). In this revised version an additional 16 ACT exercises and 8 metaphors are added, divided over all 6 instead of 3 ACT components (in line with the feedback for more exercises and variation). Also the number of beeps are lowered from 10 to 8 per day (thereby lowering the amount of metaphors offered and restricting to two different metaphors per day). Furthermore, the differences between the regular ACT exercises and ACTion exercises are more articulated. When participants activate the training menu, a question is added that helps to guide them to the exercise that is most appropriate for that particular moment. For example, when participants answer yes to the question “Do you experience unpleasant thoughts, feelings, or sensations at this moment?” they are guided to the ACTion exercises that are specifically designed to deal with negative experiences in the present moment. If answering no, participants are guided to general ACT exercises. Finally, the setup of the intervention is changed from sequential (first ACT training, than mHealth training) to synchronized (ACT training and mHealth training combined), and the training is extended from a 4-week to a 7-week training.

Finally, a preliminary examination of the effectiveness of the ACT-DL was performed. These results showed no short-term effect immediately after termination of the ambulatory ACT-DL on top of the effect of the inpatient ACT group intervention. We cannot rule out that subtle add-on effects of the ACT-DL were obscured by the impact of the transition from an intensive inpatient treatment to an ambulatory treatment. This effect was apparent in both ACT-DL and control participants. It is also possible that a ceiling effect, due to the intensive inpatient treatment, hampered measurement of additional improvement. Additionally, only short-term effects were investigated since there were no follow-up data available to assess the effectiveness of the ACT-DL in the long term. Differences may emerge at a later stage as it has been shown that effects of acceptance-based interventions can increase over time [41]. A recent RCT(randomized controlled trial) from Lappalainen [42] showed significant differences at 18-month follow-up.

### Limitations

Our study had some limitations. User evaluation is sensitive to recall bias. Therefore, ESM data could a valuable source of information on user experience, but a structured log was not available to provide this data. Hence, objectifying ACT practice was not possible. Also, there was no information available on the dropout rate in the control group making it impossible to compare rates. The proportion of females was higher in the ACT-DL group than the control group; female participants were apparently more willing to participate in the study or more willing to take ACT-DL into use. Overall, this study has a high proportion of females, and this could limit generalizability of results to males. A final limitation is the observational nature of the study. It is possible that the groups differed from each other on unmeasured confounders.

### Conclusions

This is the first study that uses experience sampling, fostering awareness in daily life, in combination with acceptance and commitment therapy, fostering skill-building in daily life. The study suggests that the combination not only fits theoretically but also seems to function well in practice. Another strength of this study is the use of a heterogeneous clinical sample. Therefore, the generalizability of the results may be high, suggesting that ACT-DL is suitable for a broad range of mental health problems. Effectiveness will have to be examined further in experimental settings that also address long-term effects.

## Appendix

Multimedia Appendix 1 Photos of ACT in Daily Life training with the PsyMate.

## Abbreviations

ACT: acceptance and commitment therapy
ACT-DL: ACT in Daily Life
ANCOVA: analysis of covariance
BSI: Brief Symptom Inventory
ESM: experience sampling method
FIT-60: Flexibility Index Test
MINI: Mini-International Neuropsychiatric Interview
PDA: personal digital assistant
QoL-VAS: Quality of life visual analog scale
SIDP-IV: Structured Interview for DSM Personality Disorders
DSM-IV-TR: *Diagnostic and Statistical Manual of Mental Disorders*(Fourth Edition)
UCL: Utrechtse Coping List
