# Supplementary figures and images for: Acceptance and Commitment Therapy in Daily Life Training: A Feasibility Study of an mHealth Intervention
Source: JMIR Mhealth Uhealth. 2016 Sep 15;4(3):e103. doi: 10.2196/mhealth.5437 (PMC5070582; doi:10.2196/mhealth.5437)

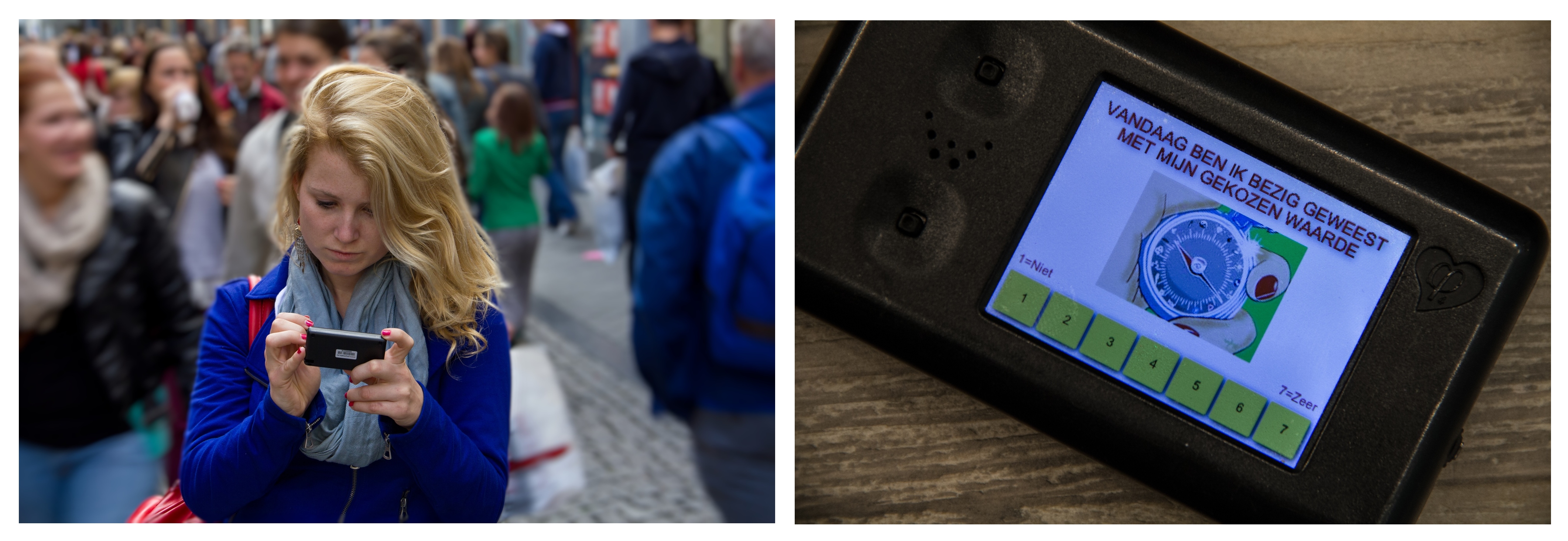

Supplement: Multimedia Appendix 1 [file mhealth_v4i3e103_app1.jpg]
